# Resources for the design of CRISPR gene editing experiments

**DOI:** 10.1186/s13059-015-0823-x

**Published:** 2015-11-27

**Authors:** Daniel B. Graham, David E. Root

**Affiliations:** Broad Institute of MIT and Harvard, Cambridge, MA 02142 USA; Department of Medicine, Massachusetts General Hospital, Harvard Medical School, Boston, MA 02114 USA; Center for the Study of Inflammatory Bowel Disease, Massachusetts General Hospital, Boston, MA 02114 USA

## Abstract

CRISPR-based approaches have quickly become a favored method to perturb genes to uncover their functions. Here, we review the key considerations in the design of genome editing experiments, and survey the tools and resources currently available to assist users of this technology.

## Genetic perturbations with CRISPR technology

The ability to edit genomes has been greatly enhanced by the adaptation of the bacterial type II CRISPR-Cas9 system into mammalian and other cell types [1–8]. This powerful technology has rapidly become a favored approach to perturb genes to probe their function. With the rapid evolution of technology and applications based on clustered regularly interspaced short palindromic repeats (CRISPRs), it is challenging for aspiring users of CRISPR technology to keep up with all the latest developments in the field and with the tools and resources available to help design and implement CRISPR-based experiments. For common applications of CRISPR-based technology in mammalian cells, we outline practical considerations in designing CRISPR-based experiments, and tools and resources available to assist in the design and execution of such experiments.

Major applications of CRISPR technologies include functional knockout (KO) of a small number of individual genes [3, 9], large-scale KO screens [10, 11], gene editing [knock-in (KI)] [2], transcriptional activation or inhibition (small scale or screening scale) [12, 13], and in vivo mouse models [14, 15]. Here, we focus mainly on reviewing strategies for editing coding genes to uncover their function. Many experimental considerations are shared across different applications, but some factors differ in their relevance or relative importance. Common considerations include delivery of CRISPR-associated protein 9 (Cas9) and guide RNAs (gRNAs) to the target cells, maximizing on-target activity and specificity, and evaluation of editing results (for efficacy, specificity). We briefly discuss the basics of CRISPR technology, then outline basic experimental design considerations and associated tools and resources, and finally highlight issues relevant for specific CRISPR applications (summarized in Box 1).

## A general description of type II CRISPR-Cas9 systems

**Fig. 1:**
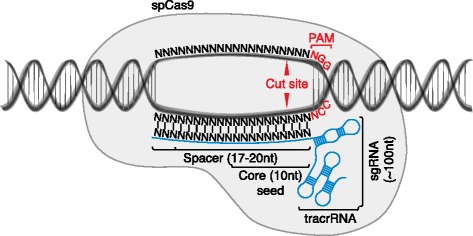
Components of the CRISPR-Cas9 system. *Streptococcus pyogenes* Cas9 (*Sp*Cas9) forms a complex with a chimeric single guide RNA (*sgRNA*) comprising a spacer that hybridizes with the genomic target site, and a scaffold RNA termed *tracrRNA* required for complex formation. The protospacer adjacent motif (*PAM*) is required for sequence specificity of *Sp*Cas9-mediated endonuclease activity against genomic DNA

As noted, CRISPR-based methods enable multiple distinct types of genetic perturbations: KO of gene function, specific edits to the genome (KI), and activation or inhibition of gene expression [16]. For all of these applications, two molecules must be introduced into each target cell — a Cas9 protein and a single guide RNA (sgRNA). These two molecules form a complex with genomic DNA (gDNA), specifically targeting DNA sites complementary to an approximately 20-base sequence within the sgRNA and neighboring a protospacer adjacent motif (PAM), the identity of which is dictated by the particular Cas9 protein employed (Fig. 1). For the most commonly used Cas9 to date from *Streptococcus pyogenes*, the optimal PAM sequence is NGG (where ‘N’ is any nucleobase). The wild-type Cas9 (wtCas9) has two endonuclease domains that produce double-stranded breaks (DSBs) in the targeted gDNA sites. Alternatively, an endonuclease-dead Cas9 (dCas9) can be used to ferry functional domains to the sequence-specified sites in the genome — for example, for transcriptional activation (CRISPRa) or inhibition (CRISPRi) at gene promoters.

**Fig. 2:**
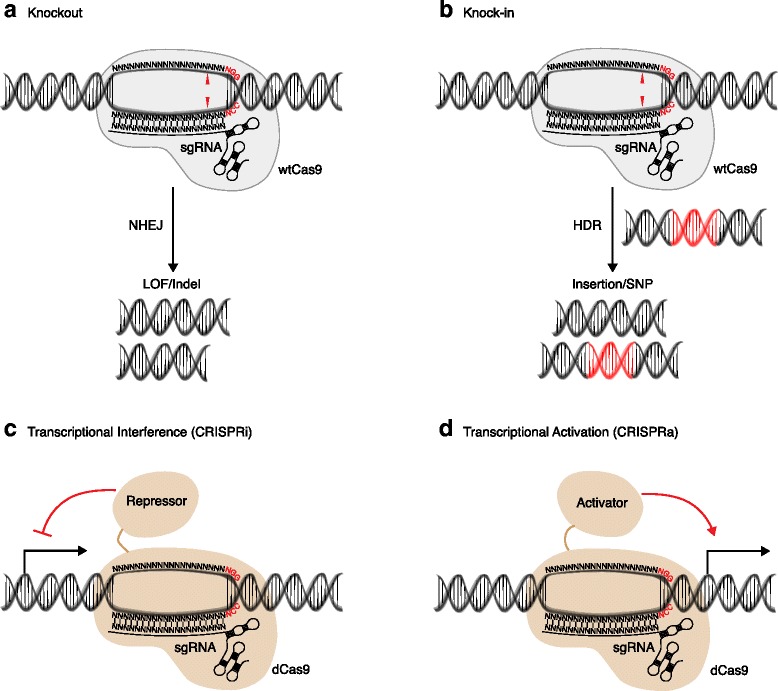
Genetic perturbations enabled by engineered CRISPR/Cas9 systems. **a** Knockout approaches generate loss-of-function (*LOF*) alleles by means of insertion/deletion (*indel*) mutations incurred by erroneous repair of DNA double-strand breaks by nonhomologous end joining (*NHEJ*). **b** Knock-in approaches aim to introduce defined mutations [e.g., an insertion or single-nucleotide polymorphism (*SNP*)] encoded by repair templates that exploit endogenous homology-directed repair (*HDR*) mechanisms. **c** Transcriptional inhibition with CRISPR interference (*CRISPRi*) employs endonuclease-dead Cas9 (*dCas9*), or transcriptional repressors fused to dCas9, to suppress gene transcription. **d** Overexpression with CRISPR activation (*CRISPRa*) employs transcriptional activators fused to dCas9 to activate gene transcription. In addition, single guide RNAs (*sgRNAs*) have been engineered that contain aptamers to recruit additional transcriptional activator complexes

For applications that modify the gDNA — for example, KO and KI — the DSBs produced by wtCas9 are subsequently repaired through endogenous DNA repair mechanisms, either non-homologous end-joining (NHEJ) or homology-directed repair (HDR) (Fig. 2). NHEJ is prone to introduce sequence insertions or deletions (indels), and can therefore produce frameshifts in open reading frames and gene loss of function. As a variety of indels are produced at each CRISPR target site in coding genes — in-frame or out-of-frame and varying in size — the resulting alleles are actually a mixture of complete functional KOs, partial loss of function, wild-type alleles, and even potentially altered (neomorphic) function. As currently implemented, the fraction of modified KO alleles typically ranges from 30–60 %, so that the cell population generally exhibits loss-of-function phenotypes. Various factors can contribute to the residual non-KO alleles, including (i) failure of Cas9 activity in individual cells — owing to a low level of Cas9 or other reasons, (ii) poor accessibility or susceptibility of the gene or target site, (iii) the NHEJ errors incurred at the targeted site frequently producing still-active alleles, and (iv) targeting multiple alleles of the same target gene sometimes being inefficient (for example, for >2 N cell lines and duplicated genomes such as zebrafish). The relative importance of the factors governing the ‘penetrance’ of KO across a cell population in different genes, target sites, cell lines, etcetera is not yet fully understood. Ideally, methods to improve, across the board, the fraction of cells or alleles converted will emerge, but, for the present, significant heterogeneity in the initial edits is unavoidable. Thus, obtaining a uniformly edited cell population currently requires picking individual cell clones for expansion. While conversion to the desired genotype is not perfectly efficient, CRISPR is nonetheless the most straightforward method to produce KOs for most applications.

To utilize HDR to edit the genome, a DNA repair template with the desired sequence modification is introduced. The HDR process that incorporates the template at DSBs is of relatively low efficiency, producing typically a single-digit or low-double-digit percentage of the desired edit in treated cells. NHEJ is more efficient than HDR, producing undesired indels in the cell population, and it will be desirable to find ways to enhance HDR versus NHEJ for KI applications. In this context, chemical inhibition of NHEJ has been demonstrated to improve the efficiencies of HDR-mediated genome editing [17, 18]. Even so, HDR remains a low-efficiency process, and, to obtain the desired genome modifications, one must isolate the low percentage (typically single digit) of single-cell clones with the desired sequence for expansion. Importantly, HDR only occurs during S and G2 phase [19], whereas NHEJ can occur at any point of the cell cycle [20]. Thus, KI approaches requiring HDR are less suited for terminally differentiated cells compared with cycling cells [21]. Conversely, KO indels created by NHEJ can be reverted to the wild-type sequence by HDR in rapidly cycling heterozygous cells, potentially slowing the accumulation of KO cells in fast-cycling cell populations.

For methods that use the CRISPR-Cas9 system to activate or inhibit gene expression, an endonuclease-dead dCas9 is used to recruit a transcriptional activating or inactivating activity to the promoter regions of genes [12, 13, 22–28]. In general, the dCas9-sgRNA system could be used as a sequence-specific binding complex to deliver, in principle, any ‘warhead’ (a functional domain, reporter, etc.) to sequence-specified target sites.

## Practical considerations and tools for the experimentalist

To obtain the best results from CRISPR-based experiments, some basic factors must be considered in the experimental design. The overall goal of CRISPR experiments is to obtain, in your preferred biological model system, high rates of the desired genome perturbation, low rates of off-target (OT) or nonspecific effects, and a good readout of the outcome. While CRISPR has proven quite powerful, the editing efficiency and specificity are not perfect, and delivery of the CRISPR system into the biological model system of interest is challenging in some systems. Therefore, it is necessary to optimize and validate experimental designs to achieve the best results.

### Delivery of Cas9 and sgRNAs and Cas9 activity

The gene encoding *S. pyogenes* Cas9 (*Sp*Cas9) can be introduced by transfection or viral transduction with a Cas9 expression construct or by direct delivery of Cas9 protein [29–34]. Furthermore, a germ-line Cas9 mouse has been generated, providing a source of animals and primary cells in which Cas9 expression is already established [35, 36]. Delivery of Cas9 by transfection can be quite efficient in many cell types; frequently employed expression vectors include pX330-U6-Chimeric_BB-CBh-hSpCas9 and lentiCRISPRv2 [3, 37, 38] (available from AddGene). In hard-to-transfect cells, including many primary cell types, transduction with a viral vector provides an alternative, using, for example, lentiCRISPRv2. Furthermore, for pooled screening applications, each cell must receive only a single or small number of sgRNAs by treatment with a mixed sgRNA pool, and hence transduction is the only standard delivery option. Delivery of *Sp*Cas9, alone or together with an sgRNA, can be achieved with adeno-associated virus (AAV), retroviral or lentiviral vectors and is challenging owing to the generally poor viral packaging and titers of the 4-kb *Cas9* gene. Whether employing transfection or transduction, Cas9 expression varies from cell to cell, and the levels also vary among cell lines. Transduced cells are typically obtained by selecting for a marker present on the Cas9 expression cassette. It is important to verify that the promoter construct employed is effective in the model of interest, and it can be helpful to grow clonal populations with empirically verified high Cas9 expression for subsequent experiments. By contrast, delivery of sgRNA oligonucleotides is relatively straightforward and can be achieved by transfection of plasmids or transduction with viral genomes driving sgRNA expression from the U6 promoter [2, 3]. Alternatively, sgRNAs can be delivered by transfection of in vitro transcribed sgRNA or chemically modified synthetic sgRNA [30].

It appears that most cell lines are amenable to CRISPR-based editing, but some cell types appear to exhibit low or no Cas9 activity even when Cas9 is expressed at high levels. In general, the factors that govern how uniformly the alleles in all the cells of a population receive the desired edit have yet to be fully teased apart and could include, for example, not only the Cas9 and sgRNA levels, but also Cas9 activity determinants such as localization, the kinetics of DSB formation, and the kinetics and fidelity of repair processes, all of which can vary across cells types. For the moment, the suitability of any particular model system of choice for CRISPR should be confirmed empirically.

A straightforward assay to assess CRISPR activity in a cell population involves transducing the cells with a cassette expressing both green fluorescent protein (GFP) and a validated high-efficacy GFP-targeting sgRNA [37] (available at AddGene). The cells are then analyzed by flow cytometry to determine the fraction of GFP-negative cells [37]. The parental line with no Cas9 should be uniformly GFP-positive, whereas a Cas9 line in which the cells are all active for CRISPR should be mostly GFP-negative. It should be noted that KO of a single GFP integrant can be considerably more efficient than targeting both alleles of an endogenous gene, so that this assay might represent a near-best-case scenario for KO rate. Additionally, the time required to achieve gene edits appears to depend on many factors, such as target gene, cell type, KO versus KI, and the levels of Cas9 and sgRNA. Generally, when feasible, it is necessary to wait a week or more following the introduction of Cas9 and sgRNA in order to accumulate edits in the targeted cells.

### Target-site selection, sgRNA design

For CRISPR-based experiments, one must select a target site to achieve the desired modification. The Cas9 protein requires a PAM adjacent to the sgRNA homology region to achieve efficient Cas9 binding and DSBs. For gene KOs, there are typically many possible PAM sites from which to choose. Different sites can yield widely varying rates of gene KO, raising the question of how to predict activity in advance. Similarly, it is obviously desirable to predict which sgRNAs will be most specific to the intended target. Research is ongoing to determine criteria that predict sites favoring high activity and specificity. Here, we describe current criteria and tools for selecting sgRNAs.

#### Design criteria for on-target efficacy

For the most-used *Sp*Cas9, the optimal PAM site is NGG or, to a much lesser extent, NAG. The NGG PAM sequence occurs on approximately every 8 bp in the human genome [3]. The relatively common occurrence of NGG sites in most genomes leaves many available target sites for *Sp*Cas9. Recently, variants of *Sp*Cas9 with altered PAM specificities have been developed [39], and some design tools offer features to accommodate user-defined PAMs (Tables 1 and 2). One such *Sp*Cas9 variant (VRER) recognizes NGCG PAM sites and was reported to exhibit greater on-target specificity than wild-type *Sp*Cas9 [39]. Additional flexibility with respect to PAM constraints can be achieved with *Cas9* genes derived from other bacterial species. For example, *Staphylococcus aureus* Cas9 recognizes NNGRR PAM sites and was demonstrated by sequencing approaches (BLESS) to exhibit greater on-target specificity compared with *Sp*Cas9, while being 1 kb smaller [40]. Although such new versions of Cas9 are emerging, most CRISPR design tools are modeled for *Sp*Cas9 and utilize NGG or NAG PAM consensus sites for sgRNA design by default.

**Table 1:** Tools for the design of guide RNAs

**Tool**	**Website**	**Reference**	**Input** ^**a**^	**Output** ^**b**^	**Throughput** ^**c**^	**Use cases**	**Genomes**	**sgRNA sequence constraints**	**Validation**	**Pros**	**Cons**	**Software**
sgRNA Designer	http://www.broadinstitute.org/rnai/public/analysis-tools/sgrna-design	[37]	Ensembl transcript IDs or nucleotide sequences	Activity-ranked sgRNA, exon, percentage protein sequence C-terminal of target site	Medium/high	Find target sequences for up to ten transcripts (small-batch mode); good for generating a lot of candidate guides quickly	Human, mouse	N/A	Meta-analysis of genome-wide CRISPR screens	Ease of use, on-target efficacy based on experiment data and validation	No OT prediction	Web/local
CRISPR MultiTargeter	http://www.multicrispr.net/	[50]	Gene/transcript ID or sequence	Activity-ranked sgRNA based on sgRNA Designer (see above). Reports percentage GC and Tm	Low	Find all target sequences for a single sequence; find all common target sequences for all transcripts for a given gene; find unique/non-unique target sequences providing multiple sequences or similar gene types	12 common genomes	5′ G or GG. Target length. PAM NGG or user specified. Paired sgRNA		Ease of use. Many options, including any PAM. Has multiple modes, separated out, that could be useful	No OT prediction. Multiple modes can be complicated	Web
Cas9 Design	http://cas9.cbi.pku.edu.cn	[102]	Input sequence or FASTA file	Target sequence and exact matches in reference genome. Percentage AT, predicted RNA folding structure for sgRNA	Low	Find target sequences for a single sequence	Ten common genomes	Target length. User-specified scaffold RNA for structural predictions		Can be used to identify potentially problematic hairpin structures in sgRNAs	No on-target efficacy prediction. No OT prediction	Web
SSFinder	https://code.google.com/p/ssfinder/	[46]	FASTA file	All potential NGG-PAM guides	High	Find target sequences for any number of FASTA sequences	N/A	None		Very simple input/output requirements. Works quickly on a small number of sequences. High throughput possible	No options. No on-target or off-target information	Python script
Cas OFFinder	http://www.rgenome.net/cas-offinder/	[103]	sgRNA	Lists OT sites with number and position of MM	Medium/high	Find comprehensive off-target information for one or more guide sequences; must run as script	Approximately 20 common genomes	Alternative PAMs, tolerance for MMs		Ease of use. Analyzes multiple sgRNAs in batch. OT sites with up to nine MMs and two bulges	Does not indicate whether OTs are in CDS. Does not account for identity of MMs	Web/local

^a^The input data are gene sequence or sgRNA sequence. ^b^The output is sgRNA sequence or off-target sites. ^c^‘Low’: input format and run times support one-gene-at-a-time or one-guide-at-a-time queries. ‘Medium’: input format and run times support small batches of genes or sgRNAs, tens to hundreds of queries. ‘High’: input format and run times support genome-scale queries

*Abbreviations:* *CDS* coding sequence,*CRISPR* clustered regularly interspaced short palindromic repeats, *MM* mismatch, *N/A* not applicable, *OT* off-target, *PAM* protospacer adjacent motif, *sgRNA* single guide RNA, *Tm* melting temperature

**Table 2:** All-in-one packages for the design of guide RNAs and prediction of off-target effects

**Tool**	**Website**	**Reference**	**Input**	**Output**	**Throughput** ^**a**^	**Use cases**	**Genomes**	**sgRNA sequence constraints** ^**b**^	**Validation**	**Pros**	**Cons**	**Software**
CCTop	http://crispr.cos.uni-heidelberg.de/	[68]	Sequences 23 to 500 bp (similar to crispr.mit input); also has single or batch mode	Scores OTs and ranks sgRNA by OTs. OT sites; position with respect to CDS. Number and position of MMs	Low	Find target sequences for a sequence or sequences (has batch mode); good for generating a lot of candidates with comprehensive on/off-target information	Approximately 15 common organisms; only a single human build available	NGG or NRG PAMs for on- and off-target. 5′ G or GG^a^. MM tolerance, total and in core. Annotated OT sites with RefSeq IDs, if applicable	On- and off-target efficacy in vitro. For one guide	Ease of use, many options. Comprehensive and easy-to-understand output	No on-target efficacy prediction. Does not account for identity of MMs. Regular mode is relatively fast. Advanced mode is slow	Web
CHOPCHOP	https://chopchop.rc.fas.harvard.edu/	[49]	Target transcript ID or sequence (raw or chromosomal position); also allows gene input	Scores OT and ranks sgRNA by off-targets, GC content, genomic map, position with respect to CDS, primers for validation, RE sites. Off-target sites 0–2 MM	Low/medium	Find target sequences for a single sequence/gene/transcript; good for generating a lot of guides for a single target quickly	Approximately 20 common organisms, plus two most recent builds for human	5′ GN, GG no TTTT, CDS, junctions, alternative PAMs; specify restrictions for position of mismatch, e.g., nine-nucleotide 5′ to PAM (seed)		Ease of use, flexible options, downloadable results	No on-target efficacy prediction. OT limited to zero to two mismatches. Does not account for identity of mismatches	Web
Crispr.mit	http://crispr.mit.edu/	[65]	Sequence or FASTA files; single or batch mode	Scores OT and ranks sgRNA by OTs, paired sgRNAs for nickase, OT sites	Low/medium	Find target sequences for a sequence or sequences (has batch mode); good for generating a lot of candidates with comprehensive on/off-target information	Approximately 15 common organisms; only a single human build available	Paired sgRNAs		Ease of use. Scores OTs for up to four MMs and provides positions. Specifies OTs in genes versus intergenic sequences	Handles short sequences 23–500 bp, although 250 bp is actual upper limit. Very slow. No efficacy metric. Does not account for identity of mismatches. Occasionally misses OT sites with no MMs	Web
WU-CRISPR	http://crispr.wustl.edu	[104]	Gene symbol or 24–30,000 bp sequence	List of sgRNA ranked by efficacy score	Low	Find target seqs based on efficacy score and absence of OT perfect seed match	Mouse and human	None		Ease of use. On-target efficacy scores based on re-analysis from [37]	OT exclusion: perfect 13-nt seed match or >85 % similarity of 20-nt sequence to exome. Doesn’t account for identity of mismatches	web
GT-Scan	http://gt-scan.braembl.org.au/gt-scan/	[69]	Sequence or FASTA file. Genomic coordinates	sgRNA, genomic sites with zero to three mismatches. Links to genome browser	Low	Find target sequences and OTs for a single sequence	Approximately 20 common organisms; only a single human build available	Many user-defined OT rules and filters. Alternative PAMs		Ease of use. Many filters to define OT rules	Has trouble finding exact matches in genome. Does not account for identity of mismatches	Web
CROP-IT	http://www.adlilab.org/CROP-IT/homepage.html	[58]	sgRNA	Lists OT sites. Scoring for OT sites. Number, position, identity of MMs. Genomic position, CDS gene name if relevant. Email results	Low	Find off-target information for a guide sequence	Mouse and human	Cas9 binding sites versus predicted cleavage sites. NGG or NNG PAMs		Ease of use. Provides gene name if OT is in exon	Analyzes one sgRNA at a time. Does not account for identity of mismatches. Email response slow	Web
Cas OT	http://eendb.zfgenetics.org/casot/	[44]	FASTA files	sgRNA and OT sites	Low/medium	Target sequences and OTs	Provided by user	Several OT rules and restrictions		Many options. Alternative PAMs, paired sgRNAs, 5′ G	Programming knowledge necessary or helpful. Does not account for identity of mismatches	Perl script
CRISPRseek	http://www.bioconductor.org/packages/release/bioc/html/CRISPRseek.html	[48]	Software package	Candidate sgRNAs with a variety of scores, dependent on parameters	High	Find target sequences and OT sites for multiple sequences; performs both nickase and paired guide design	Several common genomes	Many options. Very comprehensive in terms of design and scoring		Many options	Very laborious to both install and operate, despite having extensive documentation. Does not account for identity of mismatches	Bioconductor package in R
ZiFiT	http://zifit.partners.org/ZiFiT/	[105, 106]	Input sequence	sgRNA, OTs with zero to three MMs. Position and identity of MM. Genomic position of OT	Low	Find target sequences and OTs for a single sequence	Nine common genomes	5' G or GG		Ease of use	No on-target efficacy prediction. Does not account for identity of mismatches	Web
E-CRISP	http://www.e-crisp.org/E-CRISP/	[47]	Gene symbol/sequence	Many options for output in advanced mode; table provides sequence, three-part score (specificity, annotation of gene target regions hit, and on-target efficacy), context, number of hits	Low	Find target sequences for a single gene or sequence; performs guide evaluation as well (not tested); also cas9 nickase design for paired sgRNAs	>30 genomes	Basic mode: user defines MM tolerance, PAM sequence. Advanced mode: many options for OT specs		Lots of options, fast results, summary of all designs found	So many options — could be confusing. Does not account for identity of mismatches	Web
CRISPR Direct	http://crispr.dbcls.jp/	[107]	Transcript/genome location/nucleotide sequence	Table with target position, sequence plus context, some sequence info (GC content, Tm, poly-T), target counts plus downloads	Low	Find target sequences for a single transcript/sequence with some limited off-target info	20 common genomes	PAM type		Very fast, visual display of target sequence and OT info	No options, no on-target efficacy, OT matches limited to number of target sites with 20/12/8-mer plus PAM matches. Does not account for identity of mismatches	Web
COD	http://cas9.wicp.net	N/A	23–400 bp input sequence	GenBank file and CSV file. OT scoring. Position, identity, number of MMs. Genomic position of OT site with link to graphic	Low	Find target sequences for an input sequence. Predicts and ranks OT sites	23 common genomes	Length of target. OT stringency. NGG and/or NAG for OT		Ease of use. OT scoring	No on-target prediction. Does not account for identity of mismatches. Slow	Web
sgRNAcas9	http://www.biootools.com/col.jsp?id=103	[45]	Software package	Multiple files that include all possible designs for a given sequence plus information to filter based on cloning or on-target efficacy, OT information	High	Find target sequences for a sequence or sequences (has batch mode); good for generating a lot of candidates, with some limited on-target or OT information	User can provide any genome reference file	Can generate single or paired sgRNAs; many options for OT stringency (number of mismatches and number of offsets); several options for ease of cloning		Can generate several candidates, with some (but not a comprehensive set of) options with respect to cloning ease and efficiency and OT matching	Difficult to use. No clear on-target efficacy score. Supports only NGG PAM currently. Does not account for identity of mismatches	Local (Perl script)
DNA 2.0 gRNA Design Tool	https://www.dna20.com/eCommerce/cas9/input	N/A	Gene, locus, sequence	Display of targets; table: hit position, target sequence in context, score, overlapping gene info, number of splice variant targets	Low	Find target sequences for a single gene or sequence; performs cas9 and nickase design	Human, mouse, *Escherichia coli*, *Arabidopsis*, yeast	PAM type		Simple interface, very fast results, simple and clear output	Tied in to commercial site; output has very few data points, and unclear what is available with respect to scoring. Does not account for identity of mismatches	Web/commercial
Cas-Designer	http://www.rgenome.net/cas-designer/	N/A	Nucleotide sequence or FASTA	Target sequence and OT. Zero to two MMs and one bulge. Percentage GC. Link to Ensembl for OT sites	Low	Find target sequences. Uses Cas-OFFinder and Microhomology Predictor for OT searching	30 genome builds	Accounts for bulge MMs. Alternative PAMs		Ease of use. Fast. Accommodates bulges in OT prediction	No on-target efficacy score. OT prediction does not account for identity	Web
sgRNA Scorer 1.0	https://crispr.med.harvard.edu/sgRNAScorer/ Input	[62]	Nucleotide sequence. FASTA files up to 10 kb sequences	Target sequence with activity score. Number of OT sites with genomic coordinates	Low	Find target sequences OT searching using CasFinder. On-target activity scoring using support vector machine (SVM) model	12 common genomes	*Streptococcus pyogenes* or *S. thermophiles* PAMs		On-target efficacy score. Offers a precomputed list of target sequences for all human and mouse genes	Slow. Email output. OT prediction does not account for identity	Web
Protospacer	http://www.protospacer.com/	[51]	Many inputs. Gene ID, genomic coordinates, etc.	Target sequence with activity score. Percentage GC, OT sites, positions, identities	Medium/high	Find target sequences, OT sites, prioritize sgRNAs	Provided by user	Accounts for MMs. Alternative PAMs		On-target efficacy score based on sgRNA Designer rules (Table 1). Many options for assessing OT	Requires extensive setup. Not obvious where OTs fall with respect to CDS. OT prediction does not account for identity	Software, local

^a^‘Low’: input format and run times support one-gene-at-a-time or one-guide-at-a-time queries. ‘Medium’: input format and run times support small batches of genes or sgRNAs, tens to hundreds of queries. ‘High’: input format and run times support genome-scale queries. ^b^Options for sgRNA sequence criteria: alternative PAMs. Require 5′ G to promote PolIII-dependent transcription from the U6 promoter, or 5′ GG for in vitro transcription using the T7 polymerase. Avoid TTTT, which signals PolIII transcriptional termination.

*Abbreviations:* *CDS* coding sequence, *MM* mismatch, *N/A* not applicable, *OT* off-target, *PAM* protospacer adjacent motif, *sgRNA* single guide RNA, *Tm* melting temperature

While the NGG PAM is required for high cutting efficiency, it does not assure it. Different sgRNAs targeting NGG PAM sites produce lesions with quite different efficiencies [37, 41]. Clearly, sgRNA sequence features independent of PAM proximity are important for targeting efficiency. Insight into these other factors has been gleaned from genome-wide pooled CRISPR screens and from screens specifically designed to assess sgRNA efficacy by targeting a few easy-to-assay genes at all possible sites. One obvious variable in picking among PAM sites to generate indels and KO alleles is the position of the target site within the gene. The best results are expected for target sites in the 5′ end of coding regions in order to produce early frame shifts and stop codons. In practice, while some genes have displayed reduced KO rates when targeted at sites very near the 3′ end of the coding DNA sequence (CDS), in many cases PAM sites throughout the CDS showed similar distributions of KO efficacy [11, 37]. It is easy to see how this could vary dramatically from gene to gene. Targeting functional domains of proteins was shown to improve KO rates for one class of proteins, but generalizing this strategy would impractically require a priori structure–function knowledge for every gene of interest [42]. One trivial failure mode for KO is the targeting of an exon that is skipped in the cells being studied [37]. In the context of CRISPRa, optimal transcriptional upregulation occurs when the Cas9–transcriptional activator is targeted to the −200 bp region upstream of the transcriptional start site (TSS) [13, 22], whereas efficient transcriptional suppression by CRISPRi is achieved by targeting the Cas9–transcriptional repressor to the +100 bp region downstream of the TSS [22]. Some new CRISPR design tools now accommodate considerations for transcriptional activation and inhibition [43].

Another strong predictor of sgRNA activity is the sequence composition of the approximately 20-bp target-complementary portion of the sgRNA. First, sgRNAs containing intermediate GC content outperformed their counterparts with high or low GC content, in the context of phenotypic scoring. This observation suggests that inordinately high or low affinities of sgRNA–target-DNA duplexes negatively impact Cas9 cleavage efficiency [11, 37]. In addition to GC content, screening results indicated that a purine in the most PAM-proximal position can enhance Cas9 cutting efficacy [11]. To systematically define the rules of Cas9 on-target efficacy with respect to loss of function, Doench and colleagues [37] screened over 6000 sgRNAs tiling six murine genes and three human genes encoding cell-surface receptors. After fluorescence-activated cell sorting (FACS) of cells that had lost expression of target genes, the most effective sgRNAs were identified and examined to determine which sgRNA sequence-composition features were correlated best with efficacy [37]. In many positions of the sgRNA target sequence, certain nucleotides were significantly favored or disfavored among the most active sgRNAs, including the variable nucleotide of the NGG PAM. By *quantitatively* modeling these preferences, it was possible to predict sgRNA activity — that is, a sequence-based activity prediction model created using some of the activity data (the training dataset) successfully predicted activity of the held-out data (a test dataset). These predictions held up across different target genes, across the many sites available within each gene target, and across species (mouse or human), indicating that the observed correlations represent generalizable activity-predictive features. It was further validated that the sgRNA efficacy model showed concordance with phenotypic scores in the context of an independent genome-wide pooled screen, showing that this strategy for improving sgRNA performance translates into improved screening results [37].

#### Tools to design for on-target efficacy

How can a researcher factor current knowledge about on-target activity into CRISPR target-site selection? There are various tools now available to assist with sgRNA selection based on on-target activity considerations (Table 1). All sgRNA design tools first apply the most basic criterion for high on-target activity by identifying all PAM sites for the specified Cas9. Tools have various degrees of flexibility with respect to the genome and PAM site options; some installable software packages, such as Cas-OT [44] and sgRNAcas9 [45], flexibly permit users to input any genome of interest, but this can be an unwieldy process involving large genome sequence files and formatting to prepare input files. The user might further wish to specify certain pre-defined subsets of the genome (e.g., exomes) as a constraint for target site identification. Some tools such as SSFinder [46] simply output the complete list of PAM sites, leaving the user to dictate subsequent site selection, whereas others such as E-CRISP [47] and CRISPRseek [48] offer additional criteria to filter or rank the target sites.

Next to the PAM requirement, perhaps the most important consideration for CRISPR modifications is the position of the cut site with respect to the coding structure of the target gene. Some design tools output a graphical representation of the target gene overlayed with sgRNA sites to aid users in selecting optimal sites for genetic perturbation [49]. In addition, some tools offer options for Cas9 nickases that assist in selecting paired sgRNAs that fall within a specified distance from each other (Tables 1–2). Generally, many candidate sgRNAs fall within the desired region of the target gene, in which case an on-target efficacy prediction metric offers an additional parameter on which to prioritize among the candidate sgRNAs, such as provided by the Broad Institute sgRNA Designer or other tools that employ target scoring metrics from the Doench et al. study or elsewhere (CRISPR MultiTargeter [50], Protospacer [51]). For genomic regions in which traditional *Sp*Cas9 PAM sites might be scarce, or greater targeting specificity is required, new forms of Cas9 have been leveraged that utilize alternative PAMs. To accommodate alternative Cas9 PAM requirements, several design tools now offer options to select predefined or, in some cases, user-defined PAMs (CRISPR MultiTargeter [50]).

All of the aforementioned features relate to sgRNA function; however, design tools also incorporate options related to efficient sgRNA production (e.g., ChopChop [49]). For example, it is possible to select sgRNAs that contain a 5′ G to promote PolIII-dependent transcription from the U6 promoter, or 5′ GG for in vitro transcription using T7 polymerase. Yet another option in some design tools is exclusion of sgRNAs that contain TTTT stretches, which signal PolIII transcriptional termination.

In general, more than one sgRNA is employed for each target gene, and hence multiple designs are required. This compensates for the fact that not all sgRNAs are effective, even with the best efficacy-prediction algorithms. Furthermore, as described below, employing multiple effective sgRNAs per target is important to distinguish the consistent effects of on-target perturbation from any OT effects of individual sgRNAs. For a list of tools capable of OT prediction, see Tables 1 and 2.

#### Off-target prediction

With respect to achieving specificity, the most basic design criterion is to target only unique PAM + 20-nt sites — that is, those target sequences that occur only once in the genome. This does not, however, ensure that targeting will be perfectly specific as activity at imperfect-match ‘OT’ sites does occur. Unbiased sequencing-based approaches detected few OT mutations in the entire genome [52, 53], suggesting that the overall picture with respect to specificity is quite good. Analysis of indels induced by a single sgRNA introduced into induced pluripotent stem (iPS) cells showed only one prominent OT site [53]. By contrast, a variety of approaches suggest that rates of OT activity are not always so low, and can be quite variable among sgRNAs [54]. This makes it important to be able to predict in advance which sgRNAs will provide better specificity.

Chromatin immunoprecipitation sequencing (ChIP-seq) profiling of Cas9 binding sites suggests that homology to the PAM-proximal half of the sgRNA, sometimes termed the sgRNA core or ‘seed’ match, is sufficient to initiate Cas9 binding, but cleavage requires more extensive base pairing with the target site [55]. Thus, Cas9 can bind many genomic sites (10–1000, depending on the sgRNA), but genomic sequencing at the Cas9 binding sites demonstrates that very few of these bound sites incur indel mutations [55–57]. Another key finding from Cas9 ChIP-seq studies is that binding preferentially occurs in open chromatin, which is a factor that has been incorporated into at least one OT prediction model [56, 58]. However, the ability to routinely predict a priori or measure chromatin state across cell types is not currently feasible.

Further insight into OT effects has been obtained from direct measurement of indel rates by whole-genome sequencing [53], Digenome-seq [52], GuideSeq [59] and high-throughput genome-wide translocation sequencing (HTGTS) [60], revealing additional complexities associated with CRISPR specificity [61–64]. The Guide-seq approach suggests wide variability in the frequency of OT mutation rates produced by different sgRNAs. In a test of 13 sgRNAs, one had zero detected OT DSB sites, and the others had variable numbers of OT sites, ranging up to approximately 150 sites [59]. The same study also found that short 17-nucleotide to 18-nucleotide sgRNAs exhibited greater specificity while maintaining similar efficacy compared with 20-nucleotide sgRNAs [59]. Importantly, inspection of the identity of OT sites indicated that the sites most susceptible to imperfect-match OT activity and indel production are not readily predicted by computational methods or ChIP-seq binding data [59].

Given that CRISPR systems can be highly selective, but that sgRNAs do nevertheless show some variable levels of OT activity against imperfect-match sites, how can one design sgRNAs to minimize these OT effects? Currently, the ability to predict OT liabilities is quite limited, but recent studies suggest that better OT predictions might be possible. In general, *Sp*Cas9 cleavage efficiency is more sensitive to mismatches in the sgRNA core (or seed) sequence compared with mismatches in the 12-nucleotide region at the 5′ end of the sgRNA [59, 65]. However, there are clear exceptions to this generalization. Not all DNA–sgRNA mismatches have an equivalent impact on activity even within the core region or outside the core region; both the specific base pairings and the specific mismatch positions matter for activity [41, 65, 66]. OT prediction tools have employed heuristics such as mismatch counts for the sgRNA or within the core region of the sgRNA. Better predictions will depend on improved experimental characterization and modeling of all the factors driving specificity, including the positions and base identities of mismatches in potential OT sites [65–67].

#### Tools for OT prediction and scoring

Currently, CRISPR design tools typically use simple mismatch counts to predict OT liability. As noted above, these approximations will presumably be replaced with more refined predictions as the large systematic datasets and modeling needed to predict OT activity emerge. Several tools that use a mismatch-counting heuristic to search for potential OT sites, identifying all sites in the genome that align to a candidate sgRNA with fewer than *n* mismatches, provide flexibility for the user to determine their own criteria for utilizing mismatches in the prediction of potential OT sites (CCTop [68] and GT-Scan [69]), for example, by specifying a core ‘seed’ sgRNA region within which mismatches are assumed to be effective at blocking activity. It is important to note that most of these tools discount all sites with non-NGG PAMs despite the observation that alternative PAM sites, such as the NAG site for *Sp*Cas9, can sometimes preserve high levels of activity. While it is not recommended to target generally less-active NAG PAM sites, such sites should not be ignored as potential OT liabilities. Another key consideration for specificity scoring is the relative importance of off-targeting in different regions of the genome. For example, potential OT sites in coding regions could be of greater concern than those in intergenic regions, and some design tools permit overweighting OTs within coding genes or ignoring intergenic sites entirely. Based on the currently available design tools, a reasonable prioritization of sgRNAs for specificity in *Sp*Cas9 systems could be based on the heuristic: first, avoid perfect matches aside from the target site, including matches with the alternative NAG PAM; and, second, minimize the number of OT sites (in exons) that have a perfect match to the core ‘seed’ region of the sgRNA and fewer than three mismatches to the 5′ non-core 10-nucleotide region. Very recently, better-powered quantitative specificity-prediction models have been developed from large datasets of off-targeting frequencies for many thousands of sgRNAs [66].

Some of the tools listed in Tables 1 and 2 perform OT (mismatch) site searches; however, users should be aware that many of the algorithms used (most commonly Bowtie) are not comprehensive at finding mismatch sites: they do not reliably detect all sites with the specified number of mismatches. In particular for 2+ mismatches, Bowtie can miss a substantial fraction of sites without warning, and the fraction of sites recovered can vary in an unpredictable manner, depending on input parameters. Beyond this widely unappreciated problem in implementing OT scoring, as noted above the criteria used by most current tools to predict OT liabilities are not well-supported by empirical data, as is evidenced by the various user-definable options for these OT searches — for example, the number of mismatches allowed, core ‘seed’ region specification and different OT genome subregions. Avoiding sgRNAs with perfect OT matches in the genome is clearly wise, but otherwise current mismatch-detection OT prediction tools are generally of unknown value for improving specificity. Better quantitative models and validation are emerging and will presumably be incorporated into the next generation of design tools.

### Experimental evaluation of on- and off-target activity and clonal selection

**Fig. 3:**
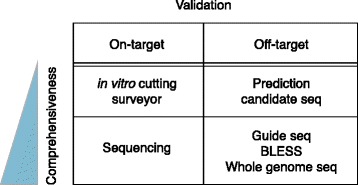
Summary of experimental options for validating CRISPR edits at the target site and off-target sites, highlighting the varying degrees of comprehensiveness that can be achieved

As with all gene-perturbation technologies, various types of validation are needed to confirm the relationship between the perturbed gene and the phenotype, and to understand the observed phenotype and its mechanism. For CRISPR-based results, one useful validation experiment is to assess the genotype of the modified cells at the intended target site, and with respect to OT effects elsewhere in the genome. Numerous approaches have been employed (Fig. 3), and determining which ones to use and the degree of validation necessary can be challenging. Validation practices are currently far from standardized, but we will outline below some of the options and the key considerations in choosing a practical path to validating the link between the gene targeted for perturbation and the observed phenotypes.

#### On-target editing confirmation

A common approach for evaluating on-target modifications is the Surveyor assay [3, 14]. Implementation is quite straightforward and involves PCR amplification of the modified and unmodified target site, followed by denaturation and annealing of the PCR products. Addition of the Surveyor nuclease then specifically cleaves double-stranded DNA (dsDNA) at mismatch sites created by indels. Under optimal conditions, this approach facilitates estimation of CRISPR indel frequency. While the strength of Surveyor assays is their rapid and simple workflow, for many target sites some level of custom optimization is required to achieve good results, and the sensitivity and quantitative accuracy are limited. Moreover, nuclease assays do not reveal the frame of indels relative to the coding sequence and cannot predict loss-of-function rates. Another approach for determining on-target cleavage efficiency employs an in vitro cutting reaction that again uses the target PCR amplicon, but combines it with transcribed sgRNA, and recombinant Cas9. While this assay is extremely sensitive, it does not reliably predict cutting efficiency in situ in cellular gDNA, as the in vitro reaction is vastly more efficient.

The most definitive means of determining on-target efficacy in cells is sequencing the target site. PCR amplicons derived from the target site can be sequenced by next-generation sequencing (NGS) to obtain the distribution of allele modifications. If access to NGS is limiting, an alternative can be to clone the target amplicon into a standard plasmid, transform competent *Escherichia coli* with the ligation products, and submit bacterial plates for colony sequencing. Many companies now offer services for Sanger sequencing directly from bacterial colonies. Importantly, sequencing approaches allow for quantitative determination of indel frequencies and out-of-frame mutations. Furthermore, programs such as ‘tracking of indels by decomposition’ (TIDE) have been developed to assist users in PCR primer design and downstream sequence deconvolution of CRISPR target sites [70].

#### Empirical OT specificity assessments

In principle, the experimenter could assess OT mutations for each sgRNA by sequencing genome-wide. In practice, the required high-coverage sequencing is impractical. The GuideSeq-type alternatives described above provide a more focused look at OT DSBs, but they too are impractical to perform on more than a small number of sgRNAs. Furthermore, while there is evidence that these methods can be quite thorough, it is hard to preclude false-negative blind-spots in their OT detection.

How can a CRISPR user identify OT effects in a practical manner in gene-function experiments? Most importantly, one should employ multiple distinct sgRNAs to target each gene. On-target effects should exhibit phenotypic concordance among different sgRNAs targeting the same gene, whereas the likelihood that relatively rare OT events will overlap among distinct sgRNAs is very small. Therefore, provided that the background rate of scoring by negative controls is low, a phenotype produced by multiple sgRNAs targeting the same gene can be ascribed to on-target activity. If target cells are to be subcloned, multiple such clones and controls should be produced so that their behaviors in experiments can be compared. A gold standard to determine whether a phenotype was caused by loss of a specific gene is to perform a rescue experiment. Specifically, introduction of cDNA encoding the target gene and mutated at the sgRNA target site should rescue the observed phenotype of a KO, provided that the phenotype is reversible and that the ectopically expressed cDNA faithfully recapitulates the gene activity.

To investigate OT mutations of individual sgRNAs, a common approach is to predict a list of likely OT sites based on sequence homology between the genome and sgRNA and then sequence these regions. As discussed above, many design tools facilitate these types of predictions, but these predictions are only as accurate as the data they are based upon, which is currently quite limited, and so the candidate site list can have high false-positive and false-negative rates. As many relevant OT sites can be overlooked, this approach is no substitute for experimental validation of sgRNAs. Such predictions can be useful for a priori selection of sgRNAs to maximize the odds of obtaining target-specific phenotypic results or the desired engineered cell clones. Specificity can also be increased by employing the paired sgRNA Cas9 nickase or FokI-chimera approaches [71, 72], although these approaches also reduce on-target efficacy and still do not guarantee perfect specificity.

Looking ahead, new versions of Cas9 or other RNA-guided nucleases (RGNs) will continue to improve the specificity of genome engineering, but experimental confirmations of specificity will still be needed. Rescue experiments and the use of multiple independent sgRNAs are the most straightforward approaches, but in some cases it can be worthwhile to empirically assess the specificity of individual ‘high-value’ sgRNAs. For example, for low-throughput experiments to generate model cells or mice that go through clonal selection, the selected clones can be assessed not only for definitive on-target modifications but also based on OT site assessments. Recent advances have provided options, but their cost limits their application to small numbers of sgRNAs. As noted above, relatively unbiased identification of OT sites can be achieved in cells by monitoring integration of exogenous DNA elements into Cas9 cleavage sites (reviewed in [73]). Recovery of the genomic coordinates of these integrants is then determined by sequencing. For example, integrase-defective lentiviral genomes [67] will primarily integrate into Cas9 cleavage sites. GuideSeq [59] and BLESS [40] approaches employ short dsDNA elements to tag DSBs created by Cas9 and rely on mapping these known DNA sequences within the context of the entire genome.

## CRISPR use cases: application-specific considerations for experimental design

### Functional knockout of individual genes

The knockout of protein function for individual genes has been a powerful tool to determine the functional role of a gene in cell-based or in vivo models [9, 74, 75]. In this approach, a cell, tissue or animal model is assayed for phenotypic changes following the selective knockout of one or more genes. CRISPR has arguably become the go-to gene-perturbation technology to evaluate gene function, and CRISPR-based gene phenotyping has become an accepted standard for confirming gene function hypotheses. Before CRISPR technology, a workhorse for mammalian loss-of-function experiments was RNA interference (RNAi), but CRISPR approaches are now favored over RNAi for many or most applications, mainly owing to its dramatically improved target specificity. In addition to its improved specificity, CRISPR can provide complete functional knockout, which has the potential to generate stronger and more-uniform phenotypes than might arise from the varying degrees of incomplete loss of function achieved by RNAi. It should be noted that RNAi represents a fundamentally different type of gene perturbation than genomic DNA modifications, and this might, in some cases, offer important advantages (e.g., if reductions in the levels of transcripts more accurately model the biology of interest), but, for many experiments, CRISPR has supplanted RNAi approaches.

For small-scale gene KO experiments, the basic three issues of (i) reagent delivery and CRISPR activity in the cells of interest, (ii) efficiency of the desired edit(s), and (iii) specificity are all important. As in current CRISPR implementations the per-cell rate of CRISPR KO typically ranges from 30–60 %, it is not possible to produce genetically uniform cells without a step of single-cell cloning to isolate and identify lines that have been modified in the desired manner. As single-cell cloning is unavoidable to obtain uniformly edited cells and requires considerable effort, it is highly desirable to achieve high CRISPR efficiencies in order to minimize the number of clones needed to obtain the desired target-site modifications. How does one contend with OT effects? Again here, cell-to-cell heterogeneity is a problem, and the cost and effort associated with fully characterizing all possible OT modifications in every cell clone are impractical. A standard strategy has been to produce multiple distinct cell clones employing several distinct sgRNAs and target sites for each gene of interest. If these clonal lines all exhibit a concordant phenotype, one can assume it is due to the common on-target perturbation rather than OT effects that would generally differ among clones, especially if different sgRNAs were employed. A minimum of three effective sgRNAs per gene is recommended. The benefits of obtaining multiple good clones places an even higher premium on good design to minimize the clone picking required. When targeting a single or very small number of genes, it is practical to curate the sgRNA selection process manually and to factor in gene-specific knowledge for each gene to optimize the on-target site selection. This permits more flexibility than for larger-scale CRISPR applications for which computational tools must be fully automated and fast enough to evaluate hundreds or thousands of genes.

### Large-scale KO screens

An increasingly common application of CRISPR-Cas9 technology is to functionally evaluate hundreds, thousands or all genes in the genome by a high-throughput screening approach. Genome-wide and genome-scale pooled screens have been successfully executed [10, 11, 76–80]. Particularly exciting with respect to these screens is the frequency of ‘multiple-hit’ genes for which most or all of the sgRNAs score strongly. In analogous RNAi screens, much lower concordance is observed among short hairpin RNAs (shRNAs) or small interfering siRNAs (siRNAs) targeting the same gene [10]. Furthermore, the validation rate of hits from these early CRISPR screens appears to be generally quite high (albeit with relatively few examples thus far), supporting the notion that these reagents will generally yield far more accurate hit-lists than RNAi.

Pooled screens require that cells with the hit phenotype can be enriched or depleted within the screened cell population. This is feasible for phenotypes that are distinguishable using FACS or by proliferation–viability (‘selections’). To perform such screens, a cell population is treated with a pooled viral library carrying many different sgRNAs. The cell population is transduced at low titer such that each cell receives a single sgRNA to knock out a different gene in each cell. At the end of the screen, genomic DNA is harvested from the hit-enriched cell population (e.g., a population that has been subjected to FACS for the hit phenotype), and PCR-sequencing is used to determine which sgRNAs were enriched among the hit cells and, therefore, by inference the list of genes whose KO produces the phenotype. CRISPR pooled-screen publications provide detailed descriptions of the methods employed [10, 11, 76]. Here we highlight several key considerations for design of sgRNA pooled screens.

To deliver Cas9 activity to the cell population to be screened, a Cas9 stable cell line can be established first, and the sgRNA pooled virus added later, or the Cas9 can be delivered simultaneously with the sgRNA. As Cas9 packages poorly and yields low viral titers, there is a practical advantage to making and expanding a stable cell line first, whereas combining the Cas9 and sgRNA in a single vector reduces the titer of the library pool, but does have the advantage of permitting single-step perturbation of the cells. As indicated above, Cas9 activity in the cells to be screened should be experimentally confirmed. The design of the sgRNA library is another key factor in screen performance. As always, sgRNAs should be designed to maximize activity and specificity. High sgRNA activity is particularly important for screens because, unlike small-scale experiments, it is not possible to select single-cell clones with the desired mutations before evaluating phenotype. The entire population of cells receiving any particular sgRNA must represent, in bulk, the phenotypic effect of that sgRNA. Thus, cells that receive an sgRNA but do not fully lose function of the target gene will dilute the apparent effect of that sgRNA in the screen. Tools for sgRNA selection for large-scale libraries must be capable of fully automated design for every gene. Multiple sgRNAs per gene are advised: first, to provide more chances for efficacy and, second, so that consistency of sgRNAs per gene can be used as a gauge of gene specificity. Popular sgRNA libraries include approximately half a dozen sgRNAs per gene (i.e., 120,000 sgRNAs for a whole genome of 20,0000 genes). Improved designs yielding higher proportions of highly active sgRNAs could reduce the number of guides employed without sacrificing the power of the library to identify hit genes. Reducing the size of the library reduces the scale and cost of the screen, permitting more cells or conditions to be tested. In cases where the cells are difficult to obtain or the screen is particularly difficult or prohibitively expensive, reducing screen scale can be not only helpful but necessary. A few publically available software tools permit the high-throughput sgRNA design and scoring required for large libraries, but those that do are generally computationally intensive and must be installed and run locally (SSFinder [46], CRISPRseek [48], sgRNAcas9 [45]).

As an sgRNA can produce heterogeneous phenotypic results for both technical (non-uniform gene modifications) and biological reasons (inherent cell-to-cell variability and stochasticity of responses), a screen must employ enough cells to ensure that each sgRNA is tested in many cells. Experience with shRNA and sgRNA screens suggests that approximately 1000–2000 cells per sgRNA (combining across all replicates) is typically sufficient, assuming that the library pool is evenly represented, with all sgRNAs present in similar abundance. In practice, for each screen, the actual number of cells needed to converge to reproducible results depends on many variables, and the scale required should be validated for each screen by comparison of independent replicates to determine whether the hit-list has converged. Several scoring schemes have been proposed for RNAi pooled screens that similarly apply to sgRNA screens. None has become standard, and they are not reviewed here. Such schemes combine the phenotypic enrichment scores from the multiple sgRNAs targeting each gene, and vary mostly in the degree to which they emphasize the magnitude of scoring (of the best sgRNA) versus constituency among the multiple sgRNAs per gene. In any case, detailed experimental validation of findings from large-scale screens is essential to confirm gene effects.

There are several contexts in which in vivo mouse pooled screens are feasible using either RNAi or CRISPR. One uses tumor xenograft models in which cancer cells are library-perturbed ex vivo and then implanted into the animal subcutaneously, orthotopically or into the blood [81]. More complex in vivo screens involve library transduction of mouse hematopoietic stem cells (HSCs) or immune cells ex vivo and then reconstituting them into the mouse through bone marrow transplant or adoptive transfer or by injection of virus into the tissue of interest for in vivo transduction [82–84]. To date, these approaches have been performed at sub-genome scale on focused sets of 20–2000 genes. For pooled screens, either in vitro or in vivo, inducible Cas9 systems for delayed gene perturbations can provide additional possibilities in screen design. Inducible systems optimized to both avoid leakiness and provide rapid efficient gene editing upon induction are in development by many groups.

Pooled screens for gene activation or inhibition are performed in a similar manner, but the library designs for such CRISPRa or CRISPRi systems differ as described above. Few such screens have been published to date, and these systems are not reviewed here, but given the advantages of modulating the endogenous gene in context versus expressing the CDS from an artificial promoter, CRISPR transcriptional modifications promise to be a popular screening approach [13, 22, 24, 26].

### Gene editing

Another mainstay application of CRISPR-Cas9 technology is to produce precise gene edits — for example, to introduce specific alleles that correlate to, and might have a causal role in, a disease phenotype. In contrast to the low-throughput and high-throughput strategies for producing gene KOs described above, this method relies on the introduction of a repair template, such that new sequence is substituted at the site of the DSB. Using these HDR-mediated edits — KI alterations — any desired sequence can be inserted to produce, for example, loss of function, gain of function or altered (neomorphic) function or to investigate variants of unknown functional status. One could engineer coding variants to model a human disease or to introduce reporter genes or epitope tags into endogenous loci [15, 17]. It is clearly advantageous to obtain specifically chosen gene edits, versus the ‘take-what-you-get’ modifications resulting from NHEJ, but it comes at the cost of reduced editing efficiency. Use of HDR currently necessitates single-cell cloning to isolate the small percentage of cells with the desired modification. As with the production of high-value KO cell lines, it is strongly advisable to produce multiple correctly modified clones generated by multiple sgRNAs to enable the consistent on-target effects to be discerned from OT effects that might be exhibited by individual clones. The required single-cell cloning and analysis make KI strategies strictly low-throughput processes, but ongoing efforts to make isolation and identification of the desired clones more efficient [85, 86], or to avoid it entirely by dramatically increasing the efficiency of the HDR process [21, 87–90], could make larger scales more feasible.

When designing KI strategies, the first consideration is the location of the DNA break. For small mutations such as single-nucleotide replacements, a DSB in close proximity to the desired site of mutation can be efficiently repaired with a short single-stranded DNA oligo encoding the desired mutation and an approximately 50-nucleotide flanking sequence on both sides [91, 92]. Introduction of large insertions such as GFP reporters can be achieved by using a longer repair template such as a targeting plasmid with 400- to 1000-bp homology arms on either side of the mutation site [15, 17, 18]. In some instances, a suitable PAM might not occur within 20 bp of the mutation site, or the sgRNA in closest proximity might have excessive OT liabilities. It is preferable to select a more specific sgRNA, even if it is over 100 bp away from the mutation site, and to use a targeting plasmid with 400- to 1000-bp homology arms to improve HDR efficiency. When using either short single-stranded DNA repair templates and longer dsDNA plasmids or PCR products, mutating the targeted PAM site is advised to prevent subsequent cleavage of modified or repaired alleles [93]. In some cases, it might be desirable to introduce several silent mutations in the repair template at the sgRNA-binding site, so as to create a distinct primer-binding site in repaired alleles to facilitate genotyping. Alternatively, introduction of silent mutations that generate a new restriction enzyme recognition sequence can be leveraged for genotyping strategies. It is important, however, that any introduced mutations in the PAM or elsewhere be silent or not disrupt splicing. Thus, it is advisable to evaluate gene expression from the modified locus, and to verify on-target integration of the repair template. Several approaches are available for detecting spurious integration of repair templates and other OT indels [94].

### Mouse models

Soon after CRISPR-mediated genome engineering was demonstrated in cultured cells, it was adapted to the generation of mutant mice [14, 15, 17, 95, 96]. Many of the same considerations for in vitro genome engineering in cells apply in vivo as well, such as the selection of target sites to maximize target efficacy and specificity against OT liabilities. To generate mice, Cas9 and sgRNA can be delivered into embryonic stem (ES) cells or injected directly into zygotes. Injection of in vitro transcribed sgRNA and Cas9 mRNA into zygotes and subsequent implantation into pseudopregnant foster mothers has produced efficient generation of KO alleles [14]. When targeting a single gene, indel mutations can be detected in a majority of the resulting mice, and two out-of-frame alleles can be observed in up to 35–40 % of mice, provided that loss of function does not compromise viability [93]. Although founder mice tend to exhibit mosaicism [97], germline transmission of modified alleles is quite efficient, suggesting that the majority of indels occur early during blastocyst development. OT mutations presumably will also be transmitted efficiently to subsequent generations [98]. By sequencing predicted OT sites in CRISPR-modified mice, investigators have documented variable OT effects depending on the sgRNA selected, but in vivo CRISPR can be quite selective, consistent with in vitro observations [14]. To try to reduce OT effects, dCas9 nickase has been employed with paired sgRNAs in vivo as well as in vitro, but unfortunately on*-*target efficiency is also reduced with this strategy. Nevertheless, it is possible to obtain up to 20 % of mice with homozygous loss-of-function alleles [93]. Even with highly selective sgRNAs, OT effects cannot be discounted when generating mice. Evaluating undesired mutations by sequencing of predicted OT sites is fairly straightforward; however, as noted, prediction of OT sites is relatively poor in both directions — it can generate an overly long list of candidate sites of which few are actually found to be modified, and yet still miss many actual OT sites. Thus, many researchers might wish to maintain breeder colonies of CRISPR-modified mice by backcrossing to wild-type mice [15, 17, 93]. There are many potential applications for such in vivo modifications, such as creation of disease models, engineering of reporter mice for in vivo assays, and even in vivo screening using pooled sgRNAs delivered, for example, to the lung or immune cells [82, 99].

## Future prospects for CRISPR-Cas9

CRISPR-Cas9 technology has emerged as a dominant technology for genetic perturbations, including editing of genome elements, modulation of transcription levels of specific genes, and engineering of model systems tagged with reporters, binding elements or other convenient handles. For research applications, it holds tremendous advantages with respect to ease of use, efficacy, specificity and versatility. There are many ongoing efforts to improve and expand CRISPR technology on multiple fronts.

One major goal is to achieve more efficient, predictable editing. If it were possible to convert every cell in a population to the desired genotype, the painstaking work of selecting and characterizing individual clones would be reduced or eliminated. This would make it feasible to engineer large numbers of clonal cell lines, or even to engineer specific alleles at a screening scale. It would also make it far more efficient to produce cells with multiple edits. One approach is to re-engineer Cas9 for desirable characteristics, including altered PAM sequences, better packaging into virus, better binding and cutting efficacy and higher specificity. The hunt is also under way for better type II Cas9 proteins [40] or other type II CRISPR proteins that might possess performance advantages, or to provide altogether new activities. The adoption of new CRISPR systems might necessitate new studies to determine their on- and off-target behavior and ideal design parameters. Experience with *Sp*Cas9 can inform strategies to determine the properties of new CRISPR systems efficiently. Heuristic rules currently employed to predict CRISPR efficacy and OT effects must be replaced with data-driven models. To truly understand the products of CRISPR systems and to predict and evaluate accurately the performance of CRISPR systems, thorough experimental evaluation of on-target modification efficacy and target-site specificity across many contexts will be required. Parallel work is under way to make transcriptional modulation easier and more predictable, building on the previous versions [12, 13, 22–24, 26–28, 100]. Transcriptional modulation approaches are being applied to non-coding as well as coding genes for which loss-of-function edits may be hard to interpret, short of deleting the entire region of gDNA [101]. Improving the modularity and versatility of the CRISPR functions that carry cargo — for example, functional domains sometimes referred to as ‘warheads’ — could make effector functions such as transcriptional modulations or targeted epigenetic changes easier to devise and use.

Given the recent history of gene-perturbation technologies, including predecessors to CRISPR for gene editing such as zinc-finger nucleases and transcription-activator-like (TAL) proteins, it is certainly possible that CRISPR will be joined by other gene-editing techniques. At this time, CRISPR-Cas9 enjoys major advantages for diverse research applications with respect to ease of use, efficacy, specificity and versatility. Continuing efforts to evaluate CRISPR technologies thoroughly with respect to their strengths and limitations in the context of different types of cells and model systems will be crucial, and research into novel variations and applications of this technology will drive new functional genomics opportunities in the coming years.

## Abbreviations

AAV: Adeno-associated virus
Cas9: CRISPR associated protein 9
CDS: Coding DNA sequence
ChIP-seq: Chromatin immunoprecipitation-sequencing
CRISPR: Clustered regularly interspaced short palindromic repeats
CRISPRa: CRISPR activation
CRISPRi: CRISPR interference
dCas9: Endonuclease-dead Cas9
DSB: Double-stranded break
dsDNA: Double-stranded DNA
FACS: Fluorescence-activated cell sorting
GFP: Green fluorescent protein
sgRNA: Single guide RNA
HDR: Homology-directed repair
KI: Knock-in
KO: Knockout
NGS: Next-generation sequencing
NHEJ: Non-homologous end-joining
OT: Off-target
PAM: Protospacer adjacent motif
RNAi: RNA interference
sgRNA: Single guide RNA
shRNA: Short hairpin RNA
siRNA: Small interfering RNA
*Sp*Cas9: *S. pyogenes* Cas9
TSS: Transcriptional start site
wtCas9: Wild-type Cas9
